# Hepatoprotective effects of *Camellia nitidissima* aqueous ethanol extract against CCl_4_-induced acute liver injury in SD rats related to Nrf2 and NF-κB signalling

**DOI:** 10.1080/13880209.2020.1739719

**Published:** 2020-03-23

**Authors:** Xiaoman Zhang, Jie Feng, Shaofeng Su, Lei Huang

**Affiliations:** School of Pharmaceutical Sciences, Guangxi Medical University, Nanning, China

**Keywords:** Oxidative stress, antioxidative, anti-inflammatory, functional food, flavonoids

## Abstract

**Context:**

*Camellia nitidissima* Chi (Theaceae) is an evergreen shrub, the leaves of which are used in many medicinal applications.

**Objective:**

To characterize the chemical composition of a 10% aqueous ethanol extract of *C. nitidissima* leaves (CNE), and to explore the protective effect of the extract against acute liver injury (ALI) in rats.

**Materials and methods:**

Male Sprague-Dawley rats were divided into six groups (*n* = 10): control and negative (0.5% CMC-Na, 5 mL/kg/d), thiopronin (20 mg/kg/d) and CNE (40, 80 and 160 mg/kg/d). All groups were treated for seven consecutive days, and then, except for the control, carbon tetrachloride was administered intraperitoneally. The biochemical parameters, mRNAs, and proteins were analyzed using enzyme-linked immunoassays kits, quantitative polymerase chain reaction and western blot. Chemical components were identified using mass spectroscopy, and the phenol and flavonoid content determined by ultraviolet spectrophotometry.

**Results:**

Pre-treatment with CNE (160 mg/kg) attenuated the pathological changes in liver tissues and decreased alanine transaminase (62 and 60%), aspartate transaminase (49 and 53%) and malondialdehyde (35 and 42%) levels in serum and liver tissues. Moreover, CNE reduced the concentrations of reactive oxygen species (55%), tumour necrosis factor-α (26%), interleukin-1β (19%) and IL-6 (19%) and blocked the nuclear translocation of p65. Pre-treatment with CNE increased anti-heme oxygenase-1 (40%), superoxide dismutase (108%) and glutathione (97%) levels through upregulating nuclear factor erythroid-2-related factor 2. Twelve compounds were detected; the content of phenols and flavonoids was determined as 34.474 ± 1.026 and 15.228 ± 0.422 mg/g crude drug in CNE, respectively.

**Discussion and conclusions:**

These results suggested that CNE is a promising agent for functional food and hepatoprotective drug against ALI.

## Introduction

The liver is a highly vascularized large organ responsible for the metabolism of carbohydrates, proteins and lipids, removal of drugs and toxins from the blood and modulation of the immune response (Kandimalla et al. 2016a). Therefore, the liver is often exposed to a variety of threats. Acute liver injury (ALI), which can derive from a variety of causes, is a life-threatening trauma with high mortality, leading to rapid degeneration of liver function (Wang et al. 2019). Severe damage to the liver can ultimately lead to liver failure (Timmer et al. 2019). Pathogenesis of ALI may be related to drugs, toxins, alcohol, metabolic diseases or chronic autoimmune hepatitis (Shi et al. 2018; Wang, Diao et al. 2018). At present, the clinical prognosis for ALI is not good, as this condition usually occurs unpredictably and deteriorates rapidly (Bae et al. 2018). Hence, for ALI, prevention is as important as an effective treatment.

How can ALI be prevented effectively? Plants used as food or medicine have great potential activity against ALI. Chinese herbal medicine is an important pharmaceutical resource with a long history and has significant potential for pharmacological development. Some plants are even used for both medicine and food. *Camellia nitidissima* Chi (Theaceae) is an evergreen shrub, distributed mainly in southern Guangxi, China and northern Vietnam, and being used as a medicine and edible tea (Wang et al. 2016). It has been cultivated successfully in Guangxi, China, and recorded by the Guangxi Zhuangzu Zizhiqu Zhuangyao Zhiliang Biaozhun Dierjuan (Guangxi Zhuang Autonomous Region Food and Drug Administration of Guangxi, China 2011). *C. nitidissima* leaves show antioxidative, antitumor, antibacterial and anti-inflammatory properties (Li et al. 2017; Hou et al. 2018; Yang et al. 2018). According to Lin et al. (2013), the extract of *C. nitidissima* leaves contains many beneficial components, including flavonoids, saponins, polyphenols, amino acids and polysaccharides. The antioxidant properties of *C. nitidissima* have been mainly attributed to the polyphenolic compounds (Wang, Ge et al. 2018).

A previous study found that *C. nitidissima* polysaccharides have a protective effect against carbon tetrachloride (CCl_4_)-induced ALI in mice (Huang et al. 2016), but the mechanism needs to be clarified. *C. nitidissima* is used as food, i.e., tea. A 10% ethanol aqueous solution of leaf extract may contain all the active constituents and is similar to the tea consumed by the general population. In this article, we performed systematic studies on the hepatoprotective activity of a 10% aqueous ethanol extract (CNE) from *C. nitidissima* leaves in rats with CCl_4_-induced ALI by examining the changes in liver function, including liver function indices and histology. The molecular mechanisms mediating the anti-inflammatory and antioxidative effects of CNE were further studied. These studies found that CNE displayed significant activity against CCl_4_-induced ALI in rats by downregulating the expression of pro-inflammatory cytokines in the nuclear factor kappa B (NF-κB) signalling pathway and activating the nuclear factor erythroid-2-related factor 2 (Nrf2) signalling pathway. Thus, CNE may be a valuable candidate agent for the prevention and treatment of ALI. The 10% *v*/*v* aqueous ethanolic extract (CNE) was analyzed by ultra-high-performance liquid chromatography quadrupole/time of flight mass spectrometry (UHPLC-Q-TOF-MS/MS) when phenols and flavonoids were found to be the effective primary constituents of CNE that prevented ALI.

## Materials and methods

### Reagents

The Folin-Ciocalteu (F-C) reagent was purchased from Sinopharm Chemical Reagent Co., Ltd. (Shanghai, China). CCl_4_ reagent was purchased from Aladdin (Shanghai, China). Gallic acid and rutin were purchased from the National Institutes for Food and Drug Control (Beijing, China). Thiopronin was purchased from Shanghai Kaibao Xinyi Pharmaceutical Co., Ltd. (Xinxiang, Henan, China). The aspartate transaminase (AST), alanine transaminase (ALT), malondialdehyde (MDA), glutathione (GSH) and superoxide dismutase (SOD) assay kits were purchased from Nanjing Jiancheng Bioengineering Institute (Nanjing, China). The rat tumour necrosis factor-α (TNF-α), interleukin-6 (IL-6) and interleukin-1β (IL-1β) enzyme-linked immunoassays (ELISA) kits were purchased from Shanghai Yuanye Bioengineering Institute (Shanghai, China). The rat reactive oxygen species (ROS) ELISA kit was purchased from Jianglaibio (Shanghai, China). Anti-phospho-NF-κB p65 (p-p65) and anti-phospho-IκBα (p-IκBα) were purchased from ABclonal Technology (Wuhan, China). Anti-IκBα (cat), anti-NF-κB p65 (p65), anti-nuclear factor erythroid-2-related factor 2 (Nrf2), anti-heme oxygenase-1 (HO-1), anti-lamin B and anti-GAPDH were purchased from Proteintech Group, Inc. (Wuhan, China). The total RNA extraction reagent was purchased from Axygen (Silicon Valley, CA).

### Preparation of CNE

*C. nitidissima* leaves were obtained from Fangchenggang, Guangxi, China, in October 2016, and identified by JF (one of the authors). The voucher specimen (20160901) was deposited in Guangxi Medical University. A 10% aqueous ethanol solution was used to extract the powdered dry leaves (500 g) at 100 °C by heating with reflux three times (2 h per time). The filtrates were combined and evaporated using a rotary evaporator. Trace solvent was removed by lyophilization with a freeze-dryer to obtain the dry extract for subsequent experiments. The yield of dried residue from the extract was 23.6%.

### The analysis of CNE with UHPLC-Q-TOF-MS/MS

The CNE was diluted with methanol/water (50%, *v*/*v*), then applied to a solid phase extraction column. Next, the solvent was removed using a rotary evaporator and the sample dissolved with acetonitrile/water (50%, *v*/*v*). Finally, the sample was filtered through a microfiltration membrane for analysis. The Thermo Fisher UHPLC procedure was used to analyse the sample on a 2.1 × 100 mm, 1.7 μm bead size C_18_ column (Waters, Milford, MA). The mobile phase consisted of solvent A, acetonitrile and solvent B, 0.1% formic acid in deionized H_2_O. Binary gradient elution was performed: 0–2 min, 5% A; 2–16 min, 5–95% A; 16–18 min, 95% A. MS analysis was carried out using a Q-Exactive mass spectrometer (Thermo Fisher Scientific) and an electrospray ionization (ESI) source. The analysis was performed using full scan mode, and the mass range was set at *m*/*z* 100–1000 in both the positive and negative ion modes. Data acquisition and processing were carried out using Xcalibur software (Thermo Fisher Scientific).

### Determination of total phenols and total flavonoids

The total phenolic content of CNE was determined by the F-C method (Li et al. 2016). We used a stock solution of 5.08 mg/mL CNE in aqueous methanol (50%, *v*/*v*). The stock solution samples (0.1 mL), F-C reagent (1.5 mL) and sodium carbonate (2 mL, 12%, *w*/*v*), were combined and the mixture brought to a final volume of 10 mL with distilled water. The mixture was allowed to stand for 60 min in the dark at room temperature before measuring the absorbance at 758 nm using a TU-1901 ultraviolet (UV) spectrophotometer (Beijing Purkinje General Instrument Co., Ltd. Beijing, China). Gallic acid was used at 0, 0.01, 0.02, 0.04, 0.08 and 0.10 mg/mL to construct a standard calibration curve.

For the determination of flavonoids in CNE, we made a stock solution by dissolving 22.23 mg of CNE in 10 mL methanol/water (50%, *v*/*v*). We used 4 mL of this stock solution, to which we added 6 mL of distilled water, 1 mL sodium nitrite (NaNO_2_; 5%, *w*/*v*), then 10 mL aluminium nitrate (Al(NO_3_)_3_; 10%, *w*/*v*), 10 mL sodium hydroxide solution (1 mol/L) and distilled water to a final volume of 25 mL. The solution was thoroughly mixed and left standing for 6 min after the additions of NaNO_2_ and Al(NO_3_)_3_. The final solution was left to stand for 15 min before measuring the absorbance at 500 nm using a TU-1901 UV spectrophotometer. Rutin was used at concentrations of 0.008, 0.016, 0.032, 0.04 and 0.048 mg/mL for the standard calibration curve of flavonoid compounds. All experiments were carried out in triplicate (*n* = 3).

### Animals and experimental treatments

Male SPF Sprague-Dawley rats (SD rats, 200 ± 20 g) were purchased from the Animal Centre of Guangxi Medical University (SYXK Gui 2014-0003, Guangxi, China). Rats were housed in a standard animal laboratory with controlled room temperature (22 ± 2 °C) and relative humidity (50 ± 10%) under a 12 h light/dark cycle. Standard laboratory food and water were available at all times. The present study was performed following the Chinese legislation and the US National Institutes of Health guidelines for the use and care of experimental animals.

After one week of adaptation, 60 rats were randomly divided into six groups (*n* = 10 per group) as follows: (I) control group and (II) negative group: 0.5% sodium carboxyl methyl cellulose (CMC-Na, 5 mL/kg); (III) positive group: thiopronin (20 mg/kg); (IV, V and VI) CNE groups: CNE (40, 80 and 160 mg/kg). All the groups were treated once a day for seven consecutive days, and 1 h after the last administration on the seventh day, group I was administered olive oil intraperitoneally and groups II to VI were administered CCl_4_ [2 mL/kg, dissolved in olive oil (50%, *v*/*v*)] intraperitoneally to induce ALI. The animals were fasted for 14 h but allowed free access to water. The rats were sacrificed, and the livers were weighed and collected. Serum samples and liver tissues were stored in −80 °C before further assays.

### Histology evaluation

Liver tissues were fixed in 10% formalin and embedded in paraffin. We cut 5-μm-thick sections, deparaffinized them in xylene and rehydrated them in graded concentrations of ethanol. We placed the sections on glass slides and stained them with haematoxylin-eosin (HE) according to the standard procedure. A laser scanning confocal microscope (Olympus, Tokyo, Japan) was used to examine the sections, and images were recorded.

### Immunohistochemical examination

The pre-treatment of liver samples for immunohistochemical (IHC) examination of p65 expression was essentially the same as for histology determination. Hydrogen peroxide (0.1%) was used to inactivate the endogenous peroxidase activity in the liver sections. After blocking the sections with bovine serum for 30 min, we incubated them with anti-p65 polyclonal antibody p65 (1:2000) for 12 h, then incubated with a secondary antibody for 1 h. Finally, we counterstained the liver sections with haematoxylin. We performed the microscopic observations using a laser scanning confocal microscope. We calculated the area of p65 expression by Image-Pro Plus 6.0 (Version X, Media Cybernetics, Silver Springs, MD).

### Determination of biochemical parameters

Liver tissues were weighed and homogenized in Tris-hydrochloride. The homogenate was centrifuged by 3500 *g* at 4 °C for 15 min to collect the supernatant for analyzing the levels of GSH, SOD, AST, ALT and MDA, and the levels of AST, ALT, and MDA were also assayed in serum using commercial kits according to the manufacturer’s instructions. TNF-α, IL-6 and IL-1β in serum and ROS in liver tissues were detected by ELISA kits.

### Western blot analysis

Nuclear, cytoplasmic and total proteins were subjected to electrophoresis on 10% sodium dodecyl sulfate polyacrylamide gels and then transferred to PVDF membranes (Thermo Fisher Scientific, Millipore, MA). After using 5% fat-free milk to block non-specific binding, we incubated the membranes with primary monoclonal antibodies against Nrf2 (1:1000), HO-1 (1:1000), p-p65 (1:1000), P65 (1:1000), p-IκBα (1:1000), IκBα (1:1000), lamin B (1:1000) and glyceraldehyde 3-phosphate dehydrogenase (GAPDH; 1:1000) for 12 h at 4 °C. The membranes were then incubated with fluorescence-labeled anti-rabbit immunoglobulin G (1:10000) for 1.5 h at room temperature. Images were captured using an Odyssey Infra-red Imaging System v3.0.16 (LI-COR Biosciences, Nebraska, NE) and quantified by densitometry scanning using ImageJ Analysis software (National Institutes of Health, Bethesda, MD). Experiments were repeated three times (*n* = 3).

### Quantitative polymerase chain reaction (qPCR) analysis

The selection of primers and probes for Nrf2, HO-1, TNF-α, IL-6, IL-1β and GAPDH was performed using Primer Express software (PE Applied Biosystems, Foster, CA). The forward (F) and reverse (R) primers and probes were as follows: Nrf2 (F): 3′-CAGCCATGACTGATTTAAGCAG-5′, Nrf2 (R): 3′-CAGCTGCTTGTTTTCGGTATTA-5′; HO-1 (F): 3′-ACAGCTTGCCCCAGGATTTG-5′, HO-1 (R): 3′-GTACAGGGAGGCCATCACCA-5′; TNF-α (F): 3′-GCATGATCCGAGATGTGGAACTGG-5′, TNF-α (R): 3′-CGCCACGAGCAGGAATGAGAAG-5′; IL-6 (F): 3′-AGGAGTGGCTAAGGACCAAGACC-5′, IL-6 (R): 3′-ATCTCACAGCAGCATCTCGACAAG-5′; IL-1β (F): 3′-ATCTCACAGCAGCATCTCGACAAG-5′, IL-1β (R): 3′-CACACTAGCAGGTCGTCATCATCC-5′; GAPDH (F): 3′-AAATGGTGAAGGTCGGTGTGAAC-5′, GAPDH (R): 3′-CAACAATCTCCACTTTGCCACTG-5′. Thermal cycling conditions were an initial 2 min at 50 °C, 2 min at 95 °C, followed by 15 s at 95 °C and 1 min at 60 °C as recommended by the manufacturer, with 40 cycles of amplification. The results were calculated using the 2^−ΔΔCt^ method.

### Statistical analysis

The experimental data are presented as mean ± standard deviation (SD). One-way analysis of variance (ANOVA) with the Duncan test was used to analyze the statistical significance among the groups. A *p* < 0.05 was considered to be significant. All statistical analyses were carried out using SPSS 16.0 (SPSS, Chicago, IL).

## Results

### Identification of the compounds in CNE

**Figure 1.**
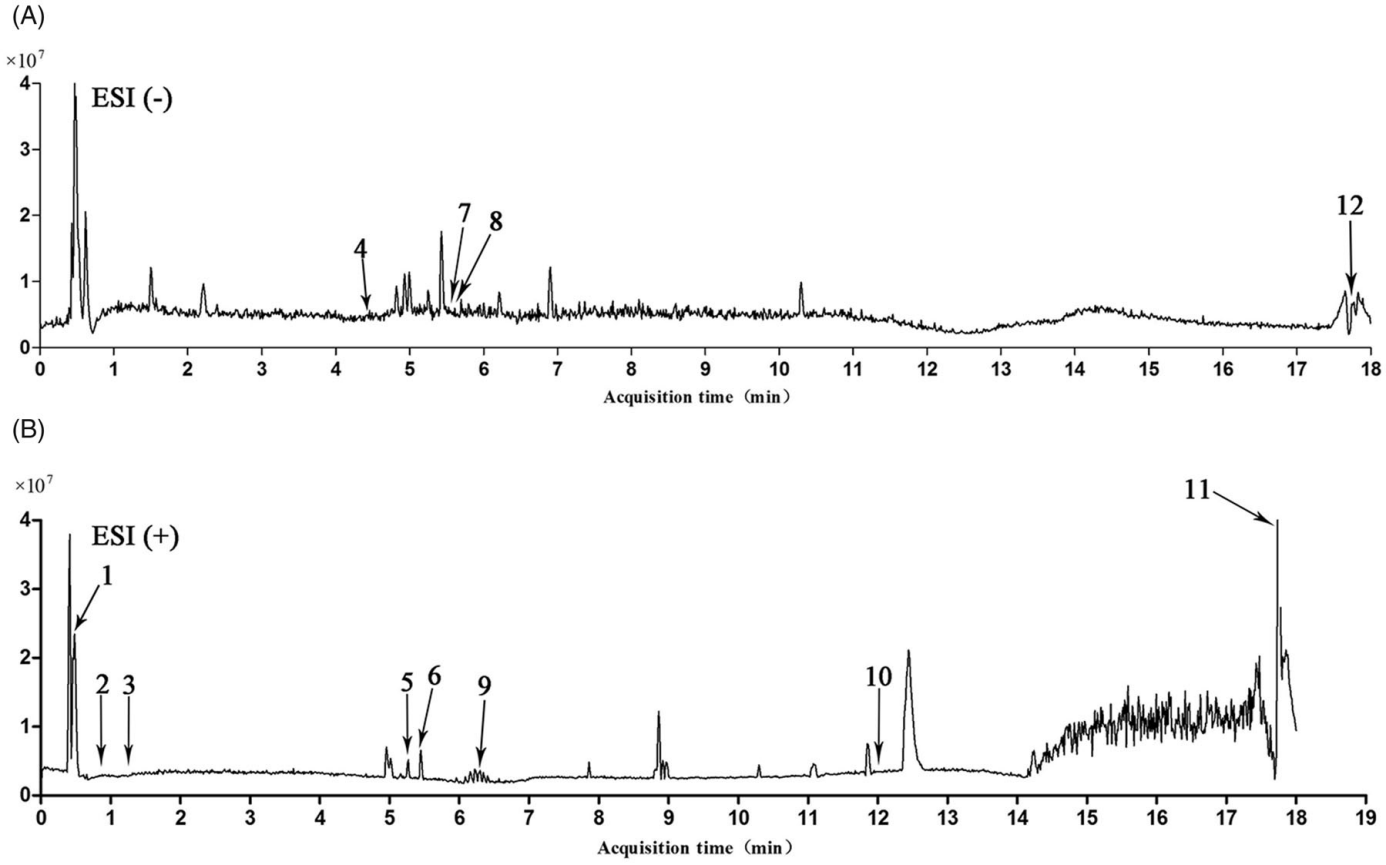
Complete ion chromatograms of CNE in (A) negative and (B) positive ion mode.

The chemical composition of CNE was analyzed using UHPLC-QTOF MS/MS; for chemical structures, see Figure 1. The main components of the CNE were identified from the mzCloud Search, Metabolika Search and ChemSpider Search (Table 1). Twelve components were identified through the matching of mass-to-charge-ratios, fragments and retention times with reference standards. l-Glutamic acid (**1**) and l-phenylalanine (**3**) are amino acids; l-phenylalanine (**3**) is one of the eight amino acids essential for humans. Pyridoxine (**2**) and nicotinic acid (**11**) are water-soluble vitamins and members of the vitamin B family. Quercetin (**5**), aromadendrin (**6**), taxifolin (**7**), rutin (**8**) and phloretin (**9**) are flavonoids. Rhein (**4**), garcinol (**10**) and (-)-epicatechin (**12**) are phenols.

**Table 1.** UHPLC-QTOF-MS/MS data for the identification of components from CNE.

No.	*t*_R_ (min)	Formula	Ion	Experimental *m*/*z*	Calculated *m*/*z*	Fragment	Identification
**1**	0.49	C_5_H_9_NO_4_	[M + H]^+^	148.0605	148.0531	84.0451	L-Glutamic acid
**2**	0.93	C_8_H_11_NO_3_	[M + H]^+^	170.0810	170.0739	151.9496, 133.9267	Pyridoxine
**3**	1.25	C_9_H_11_NO_2_	[M + H]^+^	166.0860	166.0789	120.0810, 105.9633, 84.9603	L-phenylalanine
**4**	4.41	C_15_H_8_O_6_	[M − H]^−^	283.0863	283.0320	239.0900	Rhein
**5**	5.25	C_15_H_10_O_7_	[M + H]^+^	303.1413	303.0426	287.1409, 221.0268, 153.0183	Quercetin
**6**	5.42	C_15_H_12_O_6_	[M + H]^+^	289.0701	289.0633	271.0600, 195.0288 153.0182	Aromadendrin
**7**	5.55	C_15_H_12_O_7_	[M − H]^−^	303.0513	303.0581	285.0397, 275.0550, 259.0605, 241.0496, 178.9974	Taxifolin
**8**	5.56	C_27_H_30_O_16_	[M − H]**^−^**	609.1450	609.1531	343.0444, 301.0268	Rutin
**9**	6.31	C_15_H_14_O_5_	[M + H]^+^	275.0894	275.0840	169.0496	Phloretin
**10**	12.07	C_38_H_50_O_6_	[M + H]^+^	603.4423	603.3601	411.1801, 343.1176	Garcinol
**11**	17.74	C_6_H_5_NO_2_	[M + H]^+^	124.0871	124.0322	80.0501, 78.0348	Nicotinic acid
**12**	17.75	C_15_H_14_O_6_	[M − H]**^−^**	289.0890	289.0790	245.0004, 178.8637, 136.8707	(-)-Epicatechin

### Determination of total phenols and total flavonoids

The total phenolic content in CNE was calculated as gallic acid equivalent. The standard curve equation was *y* = 0.1122*x* + 0.0249. The total phenolic content in CNE was 34.474 ± 1.026 mg/g crude drug.

The total flavonoid content in CNE was calculated in terms of rutin equivalent. The standard curve equation was *y* = 0.027*x*. The total flavonoid content in CNE was 15.228 ± 0.422 mg/g crude drug.

### CNE decreased the liver index in rats with CCl_4_-induced ALI

**Figure 2.**
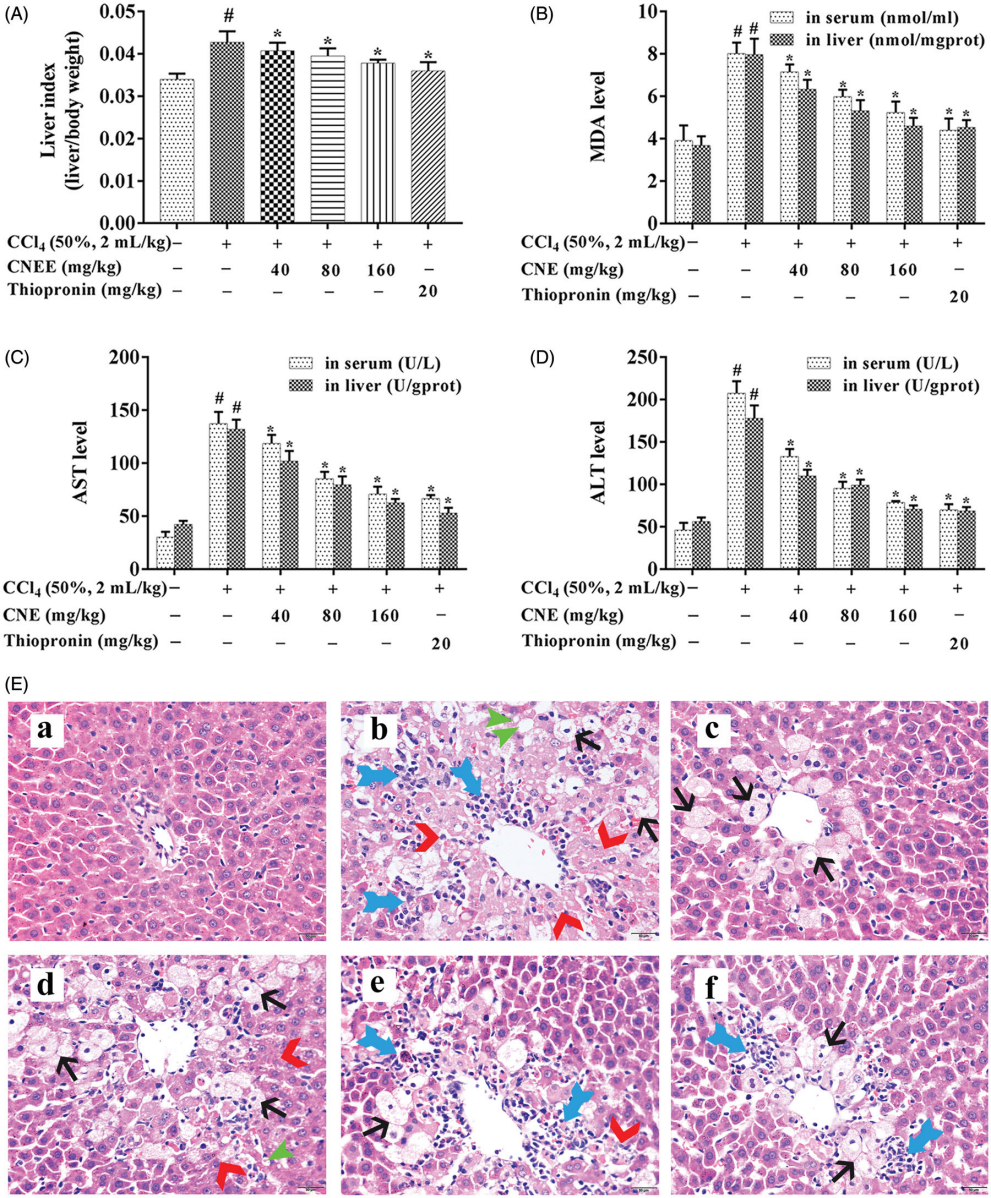
CNE decreased the liver index and the levels of AST, ALT, and MDA in rats with CCl_4_-induced ALI. Liver index (A), body:liver weight ratio in rats; the MDA (B), AST (C), and ALT (D) levels in liver and serum. The data are shown as mean ± SD, *n* = 10. #*p* < 0.05 compared to the control group; *p < 0.05 compared to negative group, for all figures. CNE attenuated the histopathological changes induced by CCl_4_ in rats with ALI (E). CNE prevented inflammation (
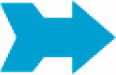
), necrosis (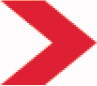), steatosis (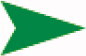), and ballooning degeneration (
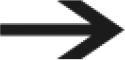
) in ALI rats. The HE staining of liver sections obtained from (a) control group; (b) negative group; (c) positive group; (d)–(f) CNE groups treated with 40, 80, and 160 mg/kg, respectively. Scale bar = 20 µm.

Figure 2(A) shows body/liver weight ratio as the liver index. The liver index was significantly increased by CCl_4_ administration in rats, but this effect was prevented by the administration of CNE in a dose-dependent manner.

### CNE decreased AST, ALT and MDA levels in rats with CCl_4_-induced ALI

The levels of AST, ALT and MDA in serum and liver tissues were detected by commercial kits (Figure 2(B–D)). In the negative group, AST, ALT and MDA levels in both serum and liver were significantly increased compared with the control group (*p* < 0.05). In the positive group (treated with thiopronin), this trend had been effectively reversed. After treatment with 40, 80 and 160 mg/kg CNE and thiopronin, AST, ALT and MDA levels were significantly decreased compared with the negative group (*p* < 0.05). The AST, ALT and MDA levels in the group treated with CNE at 160 mg/kg were close to that of thiopronin.

### CNE attenuated histopathological changes in CCl_4_-induced ALI rats

As shown in Figure 2(E), histological examination of the normal control group showed normal cellular architecture with normal hepatic cells, sinusoidal spaces and central veins. In contrast, an examination of the negative group revealed the most severe damage among the groups treated with CCl_4_: the liver sections showed massive fatty changes, necrosis, ballooning degeneration, broad infiltration of inflammatory cells and loss of cellular boundaries. Treatment with CNE (40, 80 and 160 mg/kg) resulted in the protection of the liver in a dose-dependent manner. The group treated with 160 mg/kg CNE showed significant histological improvement with a mild degree of necrosis comparable to the positive group.

### Treatment with CNE attenuated CCl_4_-induced high ROS levels in ALI rats

**Figure 3.**
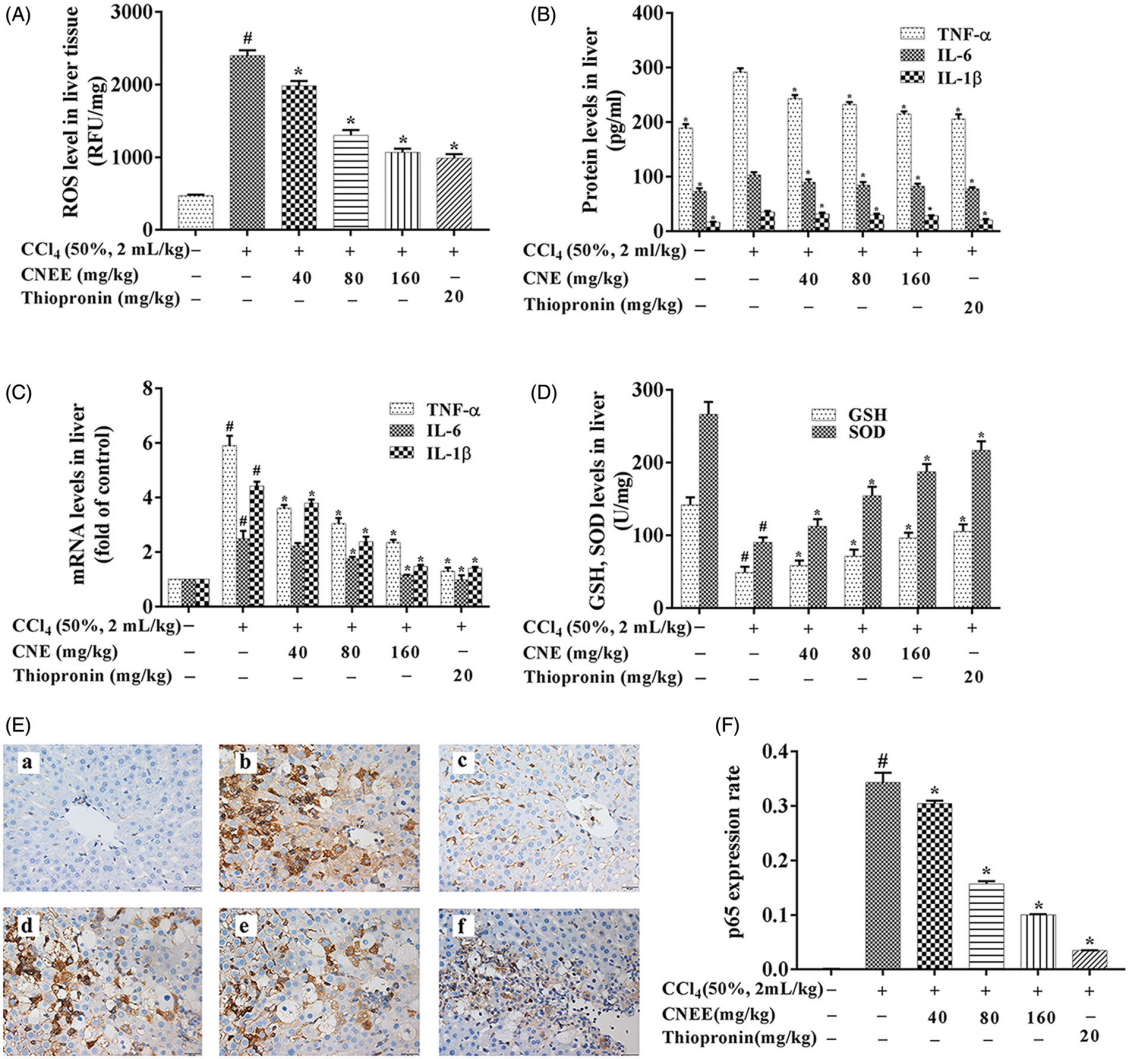
CNE mitigated oxidative stress, reduced the levels of pro-inflammatory factors and enhanced the antioxidant capacity in CCl_4_-treated ALI rats. (A) CNE attenuated the ROS levels (*n* = 10) and (B) decreased the protein levels (*n* = 10), as well as the (C) mRNA levels (n = 3) of TNF-α, IL-6 and IL-1β in rats with CCl_4_-induced ALI. The values are expressed as the fold increase of relative quantity (RQ) values normalized to the control group values (control = 1). (D) CNE increased the levels of GSH and SOD in rats with CCl_4_-induced ALI (*n* = 10). (E) The IHC examination of p65 expression: control group (a), negative group (b), positive group (c) and CNE groups (d–f): 40, 80 and 160 mg/kg. Scale bar = 20 μm. (F) The expression rate of P65 protein was calculated by the ratio of the expression area of p65 to the total number of pixels (*n* = 3). The results are represented as mean ± SD.

As shown in Figure 3(A), the level of ROS was highest in the negative group (*p* < 0.05). However, treatment with CNE (40, 80 and 160 mg/kg) or thiopronin significantly decreased the CCl_4_-induced ROS levels in the livers of rats compared with those in the negative group (*p* < 0.05).

### CNE inhibited TNF-α, IL-1β and IL-6 production in rats with CCl_4_-induced ALI

The effect of CNE on inflammatory cytokines, including TNF-α, IL-1β and IL-6, was examined by ELISA and qPCR (Figure 3(B,C)). The mRNA and protein levels of TNF-α, IL-1β and IL-6 were significantly higher in the negative group than in the control group (*p* < 0.05). However, CNE treatment significantly suppressed TNF-α, IL-1β and IL-6 mRNA and protein levels in a dose-dependent manner compared to the negative group (*p* < 0.05).

### CNE enhanced the antioxidant capacity in CCl_4_-treated ALI rats

Figure 3(D) illustrates the effect of CNE on antioxidant capacity. The CCl_4_-treated rats showed significantly decreased GSH and SOD levels compared to the untreated control rats (*p* < 0.05). Conversely, the treatment of CNE resulted in significantly increased GSH and SOD levels compared with the negative group (*p* < 0.05).

### CNE inhibited NF-κB signalling pathway in CCl_4_-induced ALI rats

**Figure 4.**
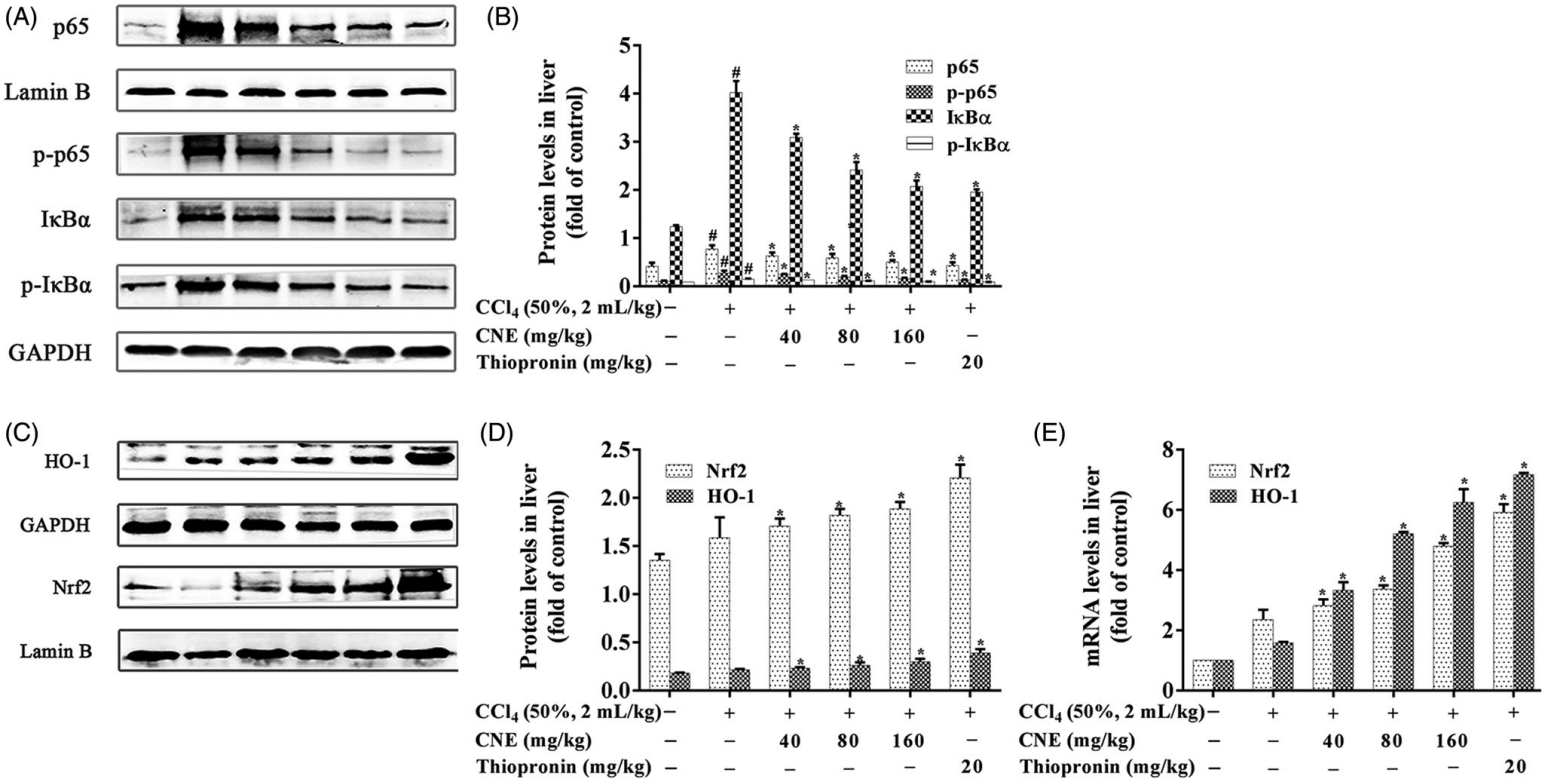
CNE inhibited the NF-κB signalling pathway in rats with ALI. (A,B) The protein levels of p65, p-p65, IκBα and p-IκBα in liver tissues. Lamin B was used as the control for p65 in nuclear protein, and GAPDH was used as the control for p-p65, IκBα and p-IκBα in cytoplasmic proteins (*n* = 3). (C,D) CNE activated the Nrf2 signalling pathway in rats treated with CCl_4_. The protein levels of Nrf2 and HO-1. Lamin B was used as the control for Nrf2 in nuclear protein and GAPDH was used as the control for HO-1 in total proteins. These values were expressed as the ratio of the optical density value of each target protein to that of its control protein. (E) The mRNA levels of Nrf2 and HO-1. The values are expressed as the fold increase of RQ value normalized to the control group values (control = 1; *n* = 3). The results were represented as mean ± SD.

CCl_4_-treated rat livers showed clearly an upregulation of nuclear p65 expression on examination by IHC (Figure 3(E,F)). On the contrary, CNE effectively downregulated nuclear p65 expression in the liver of rats with ALI (*p* < 0.05). Western blot results (Figure 4(A,B)) showed that the nuclear p65 and cytoplasmic p-p65 expression in the negative group were increased compared to the control group (*p* < 0.05). However, CNE effectively reversed this trend compared to the negative group (*p* < 0.05). In the negative group, the protein levels of both IκBα and p-IκBα showed pronounced upregulation in comparison to the control group (*p* < 0.05), but CNE administration significantly reduced expression of p-IκBα and IκBα in livers in comparison to the negative group (*p* < 0.05).

### CNE activated the Nrf2 signalling pathway in rats with CCl_4_-induced ALI

The expression levels of Nrf2 and HO-1 in livers are shown in Figure 4(C–E). Compared with the negative group, CNE-treated groups showed significantly enhanced expression of the Nrf2 and HO-1 proteins (*p* < 0.05, Figure 4(C,D)). The results of the qPCR analysis are consistent with the results of western blot analysis. In comparison to the negative group, CNE significantly promoted expression of the mRNAs of Nrf2 and HO-1 (*p* < 0.05, Figure 4(E)).

## Discussion

This study aimed to explore the effect of CNE on ALI in rats. The study proved that pre-treatment with CNE maintained the levels of ALT, AST and MDA close to normal and reduced histopathological changes in rats with CCl_4_-induced ALI. The mechanism of ALI was found to involve a complex interplay among the molecular processes involved in inflammation, oxidative stress, necrosis, apoptosis and autophagy (Tang et al. 2019). Rat liver damage caused by CCl_4_ is characterized by inflammation, the formation of trichloromethyl radicals and the overproduction of ROS, which decrease the activities of antioxidant enzymes, initiate lipid peroxidation and lead to hepatotoxicity (Yang et al. 2018). Inflammation is a natural defence response of living organisms with a vascular system to damage from factors such as pathogens, irritants or physical injury. However, ROS produced by oxidative stress can lead to excessive production and accumulation of inflammatory factors that may cause severe liver damage and even liver cancer. In daily life, various causes such as viruses, chemicals and chronic alcoholism can induce liver damage by producing large amounts of ROS (Kandimalla et al. 2016a, 2016b, 2016c). The present study showed that the ROS level was decreased by CNE pre-treatment.

Nrf2 plays a key role in the antioxidant response by controlling the transcription of various detoxifying and antioxidant enzymes to eliminate ROS. Under physiological conditions, Nrf2 is fixed in the cytoplasm via binding to kelch-like ECH-associated protein 1 (Keap1). Once attacked by ROS, Nrf2 is activated and enters the nucleus, binding to the antioxidant responsive element (ARE) and promoting phase II enzymes (such as HO-1 and SOD) and GSH expression to maintain cellular functions (Lee et al. 2019). In the present study, CNE promoted the translocation of Nrf2 into the nucleus, which resulted in an upregulation of GSH, SOD, and HO-1 expression.

When stimulated by ROS, NF-ĸB p65 becomes phosphorylated and translocated into the nucleus to regulate the expression of a wide array of genes, to govern various cellular functions such as oxidative stress and inflammation (Aliomrani et al. 2016). TNF-α, IL-1β and IL-6 have been shown to be downstream effectors of NF-κB p65 (Yang et al. 2019), and their excessive production induces the accumulation of neutrophils and releases other cytokines and chemokines (Torres et al. 2016). In the present study, the levels of IL-1β, IL-6 and TNF-α were decreased after CNE pre-treatment. In addition, IHC examination and western blot revealed that CNE inhibited IκBα and its phosphorylation and decreased the phosphorylation and nuclear translocation of p65.

In this article, 12 components in CNE were detected by UHPLC-Q-TOF-MS/MS. Among the 12 components, garcinol, pyridoxine, (-)-epicatechin, phloretin, quercetins, rutin, nicotinic acid, taxifolin and rhein showed promising pharmacological activities in the management of inflammation, oxidative stress, and liver disorders (Arauz et al. 2015; Behera et al. 2016; Ren et al. 2016; Bu et al. 2018; Roh et al. 2018; Huang et al. 2019; Lee et al. 2019; Sunil and Xu 2019).

There are some limitations to the current study. The bioactive chemical constituents would need to be purified and quantitatively analyzed. Since not all targets in the NF-κB and Nrf2 signalling pathways had been studied, further supplementation and validation are needed. Furthermore, the detection of side effects, toxicity and pharmacokinetic properties are necessary in order to fully elucidate the effectiveness of *C. nitidissima* as a preventative treatment for ALI.

Although CNE is a crude extract, it is a smaller dose than the one showing pharmacological activity as good as *C. nitidissima* polysaccharides (Huang et al. 2016). Therefore, besides polysaccharides, the main active ingredients of CNE may also include other ingredients, such as polyphenols and flavonoids. This *in vivo* study confirmed that CNE was a beneficial hepatoprotective agent by inhibiting NF-κB and activating Nrf2 signalling pathways in rats with CCl_4_-induced ALI.

## Conclusions

The hepatoprotective effects and mechanism of CNE were investigated in CCl_4_-treated rats. The levels of ALT, AST and MDA in serum and liver tissues were decreased by pre-treatment with CNE, while histopathological alterations were attenuated, and the production of ROS was suppressed. Moreover, levels of inflammatory cytokines such as TNF-α, IL-1β and IL-6 were reduced, and the phosphorylation of p65 was blocked in a dose-dependent manner. In addition, CNE treatment increased the levels of HO-1, SOD and GSH by upregulating Nrf2 in rats with ALI induced by CCl_4_. These results suggested that CNE is a promising agent for the treatment of CCl_4_-induced ALI and could serve as a candidate for the development of functional food.
